# Prevalence and antimicrobial resistance pattern of bacterial meningitis in Egypt

**DOI:** 10.1186/1476-0711-8-26

**Published:** 2009-09-24

**Authors:** Lamyaa Shaban, Rania Siam

**Affiliations:** 1 Biotechnology Graduate Program, American University in Cairo, Cairo, Egypt; 2 US-Naval Medical Research Unit-3, Abbassia, Cairo, Egypt; 3 YJ-Science and Technology Research Center (STRC), American University in Cairo, Cairo, Egypt; 4 Department of Biology, American University in Cairo, Cairo, Egypt

## Abstract

Infectious diseases are the leading cause of morbidity and mortality in the developing world. In Egypt bacterial diseases constitute a great burden, with several particular bacteria sustaining the leading role of multiple serious infections. This article addresses profound bacterial agents causing a wide array of infections including but not limited to pneumonia and meningitis. The epidemiology of such infectious diseases and the prevalence of *Streptococcus pneumoniae, Neisseria meningitidis *and *Haemophilus influenzae *are reviewed in the context of bacterial meningitis. We address prevalent serotypes in Egypt, antimicrobial resistance patterns and efficacy of vaccines to emphasize the importance of periodic surveillance for appropriate preventive and treatment strategies.

## Introduction

Emerging infectious diseases will always pose a world threat because of the continuous battle with pathogens that undergo antigenic changes to escape our immune system, and resist antimicrobial treatment. Communicable diseases are particularly challenging in developing countries because of poor-socioeconomic conditions that facilitate the spread of the pathogens and the abuse of antimicrobial therapy that results in emerging antimicrobial resistant strains, that are resistant to conventional antimicrobial treatments and possibly vaccines. This article addresses communicable diseases that are either transmitted through airborne mechanisms or contact including droplets and discharge from nose and throat. The epidemiology of three bacterial meningitis agents will be reviewed with emphasis on prevalence, antibiotic resistance and serotypes, to provide the platform for effective treatment and vaccine strategies in the region. *Streptococcus pneumoniae *is a leading causative agent of diverse infections. In Egypt, it was recently described as the leading cause of bacterial meningitis [1,2] skewing the epidemiology from *Neisseria meningitidis*, which was previously reported as the major etiological agent [3-6]. Acute respiratory infections caused by *S. pneumoniae *are frequently reported, yet more recent data are needed to form a comprehensive and updated understanding of serotype distribution and antimicrobial resistance pattern in the region [1,7]. A few studies addressed pulmonary infection from *S. pneumoniae *[8,9], with the majority of the studies addressing pneumococcal meningitis. Reports on the serotypes of isolates obtained during the studies of pneumococcal meningitis represent a fraction of all the serotypes causing different forms of pneumococcal diseases [7]. Additionally, the prevalent serotype distribution had shown variation along different studies conducted at different time intervals [7,10,11]. In order to assess effectively the epidemiology of the diseases for effective preventive and treatment strategies we need to periodically reassess the serotype prevalence. Currently the major serotypes reported in Egypt are {6B,1,19A,23F,6A} which are inadequately represented in the current 7- and 11- valent vaccine [2,7] urging the production of an effective regional vaccine. Additionally, penicillin resistance was reported by several studies at different time intervals [1,2,8,9,12], with an increase in the pattern of resistance over time; in 1993 71% *S. pneumoniae *were susceptible to penicillin [8], in 2000 63% of isolates were susceptible to penicillin [9], in 2004 51% of isolates were susceptible to penicillin [2], therefore periodic monitoring of the patterns of antimicrobial resistance is necessary to guide effective treatment [4]. In a study conducted from 1998-2004, 4% of *S. pneumoniae *(of 560) isolates conferred multidrug resistance and 50% of these were characterized as serotypes 23F, 6B, and 6A, which are the prevalent serotypes in Egypt [2]. A recent study conducted from 1998-2003 to identify the antimicrobial susceptibility and serotype distribution focused on pneumococcal isolates obtained from CSF [7]. However, isolates causing different forms of pneumococcal diseases other than meningitis were not addressed.

*Neisseria meningitidis *was for long reported as the leading cause of bacterial meningitis in Egypt, since the early studies conducted in 1965-1989 [3,5,13]. Recently it was described as the second or third leading cause after *S. pneumoniae *[1,2,4]. Earlier studies have reported serogroup A to be the predominant virulent serogroup [3,13,14]. However, few studies indicated that serogroups B [1,2,4] and C are replacing serogroup A [10,15], and this change in the epidemiology of meningococcal meningitis can be attributed to the use of the group A polysaccharide vaccine [16-18]. Unfortunately, case mortality in groups B and C were reported to be higher than A [3,10]. Sulpha-resistant *N. meningitidis *was detected since 1965 [5,6]. Due to the lack of standard criteria to evaluate the antibiotic susceptibility of *N. meningitidis*, isolates are not evaluated to determine the resistance in most studies. To the best of our knowledge, Afif *et al*., was the only study that addressed the resistance profile findings in Egypt where 86% of isolates were found resistant to co-trimoxazole and more than 40% were resistant to penicillin [2]. Most recently a molecular study investigated culture negative CSF specimens from meningitis patients to rigorously identify prevalent etiological agent, they reported an increase in detection rate of *N. meningitidis *from 16% to 23% making it the second leading cause of bacterial meningitis [2]. It was rationalized that the presence of the high number of culture negative disease in several studies conducted in Egypt may be due to the uncontrolled use of antibiotics prior to hospital admission [2,4,6,10,19].

*Haemophilus influenzae *was less implicated in meningitis in earlier studies conducted in Egypt [3,13], yet recently it was reported (serotype b) to be one of the leading causes of bacterial meningitis in children [1,2,4]. Resistance to ampicillin, chloramphenicol, ceftriaxone and co-trimoxazole were detected [1,2]. It was reported in several studies that serotype b is responsible for invasive diseases caused by *H. influenzae *[4,5,10]. However, in a study conducted in 1993 on Egyptian children below 5 years *H. influenzae *non-b was found to contribute to 43% of the pneumonia isolates, questioning the large scale use of Hib vaccine. Sparse epidemiology studies are available addressing the role of *H. influenzae *as a pathogen in Egypt [20].

The epidemiology of bacterial meningitis will be addressed in the following section by reviewing the prevalence of each of these bacteria over 39 years, and addressing antimicrobial resistance, serotypes dominance and vaccine efficacy.

## Epidemiology of bacterial meningitis in Egypt

### Streptococcus pneumoniae

*Streptococcus pneumoniae *is known to give rise to several severe infections. In Egypt it was recently described as the leading cause of bacterial meningitis [1,2,4] reflecting a change in the epidemiology of the disease where *N. meningitidis *was for a long time the main etiological agent causing bacterial meningitis [3,5,6,13]. Acute respiratory infections caused by *S. pneumoniae *require extensive reporting [1,7,8]. More recent data need to be generated pertaining to serotype distribution and antimicrobial resistance patterns, to ensure effective treatment measures. The following paragraphs address reports on meningitis caused by *S. pneumoniae *in Egypt.

#### Pneumococcal meningitis is currently the leading cause of meningitis in Egypt

Meningitis caused by *S. pneumoniae *is often referred to as pneumococcal meningitis. *S. pneumoniae *has one of the highest mortality rates amongst meningitis cases especially in patients less than one year of age [3,10,13].

Several studies were conducted between 1965 and 2004 on the epidemiology of pneumococcal meningitis, and revealed a constant rise in the number of *S. pneumoniae *meningitis in Egypt. All studies conducted before the mid 1990s demonstrated *S. pneumoniae *either at second or third place as the meningitis-causing agent in Egypt [3,5,6,13]. A longer comprehensive study ES^1966-1989 ^on 7,809 patients admitted to the Abbassia Fever hospital (AFH), reported that 7.3% of patients that suffered from meningitis were due to pneumococcal infection and the peak in the number of cases were during Jan-April. The mean age of the patients was 11.7 with 41% mortality, this is five times greater than the mortality caused by *N. meningitidis *which was the leading cause of bacterial meningitis during this period. Expectedly, 68% of the mortality cases reported were less than one year of age [3].

A one year study ES^1977-1978 ^conducted on 1627 CSF specimens obtained from two fever hospitals in Cairo revealed 350 specimens positive for bacterial infection, where pneumococci were the prevalent bacteria detected compromising 8.7% of the total specimens studied (1627) and 40.6% of bacterial positive specimens (350) with most cases reported from Jan-May. It is worth noting that the etiological agent for a large number of the specimens was not identified. The mortality rate was 44% and again mortality was mainly reported in patients less than 1 year. Serotyping by Quellung reaction identified type 1 to be the most capsular type [10].

**Figure 1:**
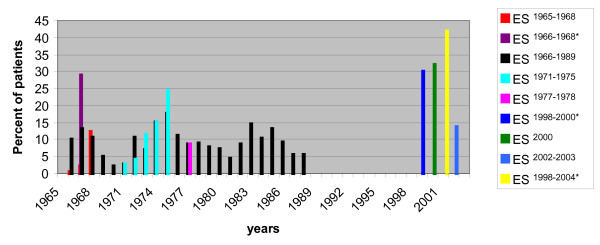
**Percentage of pneumococcal meningitis cases based on studies conducted over 39 years**. A representation of nine different studies conducted on patients diagnosed with meningitis due to different etiological agents from 1965-2004. In the study ES^1965-1968 ^on 644 cases of ABM 3% (21 patients) of the cases reported were due to pneumococcal infection [5]. ES^1966-1968 ^conducted on 187 meningitis patients *S. pneumoniae *was responsible for 29% of 123 culture-positive cases [6]. ES^1966-1989 ^conducted on 7,809 meningitis or encephalitis patients, reported that 7.3% of total cases were due to pneumococcal infection [3]. A retrospective review ES^1971-1975 ^of 1,333 patients with ABM, reported 7.9% of meningitis cases diagnosed with pneumococcal meningitis [13]. ES^1977-1978 ^revealed pneumococci was the prevalent bacteria detected [10]. Surveillance of 2047 patients with meningitis in ES^1998-2000 ^was conducted to determine the etiology of the disease in children less than 6 years. 30% of 228 patients of the cases confirmed by culture were positive for *S. pneumoniae*. However, in children older than 12 months and less than 6 years, *S. pneumoniae *was the leading causative agent [4]. ES^2000^revealed that *S. pneumoniae *compromised 32% of ABM [1]. ES^2002-2003 ^on 310 children clinically diagnosed with meningitis; 202 patients were diagnosed with ABM; 13.9% of total meningitis cases were infected with *S. pneumoniae *and 21.3% of ABM [21]. ES^1998-2004^*S. pneumoniae *was responsible for 42% culture-positive bacterial meningitis cases [2]. The asterisks represent epidemiological studies reporting an average during the entire period of the study.

In 2000 a project performed by the Ministry of health and population (MOHP) was carried out in 12 hospitals and identified *S. pneumoniae *as the leading cause of bacterial meningitis. A total of 2455 persons were suspected with acute meningitis where an overall of 223 had acute bacterial meningitis (ABM). *S. pneumoniae *was identified in 32% of patients with ABM [1]. Additionally, severe cases, based on clinical criteria of ABM were highly reported with *S. pneumoniae*, and were the etiological agent of 46.5% of the winter cases [21]. An articulate molecular study that included 14 hospitals in Egypt to determine the epidemiology of bacterial meningitis in 11,070 patients suspected with the disease during ES^1998-2004 ^identified *S. pneumoniae *to be the leading cause, responsible for 42% of the 843 culture-positive bacterial meningitis cases, and 6% of 1,784 culture-negative CSF specimens tested by PCR. This study by Afifi *et. al*., used molecular tools to address the etiological agent of the disease when conventional methods failed. In other studies when culture yields negative results the disease was described as purulent meningitis [13], without further investigation of the causative agent [2]. The high percentage of culture negative samples was reported in many studies in Egypt. This was explained by the high frequency of patients receiving on the counter antimicrobial drugs prior to professional evaluation. [2,4,6,10,19]. Figure 1 summarizes percentage of pneumococcal meningitis cases based on studies conducted over 39 years.

#### Antimicrobial resistance and treatment

Extensive studies are required to continually update antimicrobial susceptibility patterns. In developing countries the unregulated use of antibiotics is not uncommon, stressing the importance of surveillance of antibiotic resistant pathogen to guide empirical treatment. Several studies reported penicillin resistant *S. pneumoniae *[1,2,8,9,12], showing an increase in resistance pattern over time. Table 1 shows the decrease in the percentage of penicillin susceptible *S. pneumoniae *from 1993-2004 [2,8,9]. Such information on patterns of antimicrobial resistance is an effective mean to guide rational treatment [4].

**Table 1:** Antibiotic resistant *S. pneumoniae *isolates.

***S. pneumoniae***	**PEN**	**OXA**	**CHL**	**CRO**	**TET**	**SXT**	**ERY**	**-Number of isolates tested** **-Source**
ES ^1991-1993^ [8]	I = 22.4%	NA	R = 29.7%	NA	NA	I = 25%	I = 0%	-47
	R = 0%					R = 0%	R = 0%	-Blood
ES ^1997-2000^ [1]	I = 42%	NA	I = 0%	I = 4%	NA	NA	NA	-138
	R = 0.8%		R = 14%	R = 0%				-CSF and blood
ES ^1998-2000^ [4]	I = 52%	I = 0%	I = 0%	I = 0%	I = 21%	I = 14%	NA	-29
	R = 0%	R = 6%	R = 2%	R = 0%	R = 41%	R = 2%		-CSF
ES ^1999-2000^ [9]	R = 37%	NA	R = 18%	R = 16%	NA	R = 37%	R = 55%	-51
								-non-blood
ES ^1998-2003^ [7]	I+R =	NA	I+R =	I+R =	I+R =	I+R =	I+R =	-205
	50%		9%	6%	52%	59%	11%	-CSF
ES ^1998-2004^ [2]	I+R = 49	NA	I+R =	I+R =	I+R =	I+R =	I+R =	-206
	%		9%	6%	52%	60%	11%	-CSF
ES ^2003-2005^ [12,22]	R = 30%	NA	NA	NA	NA	NA	R = 25%	---------

The percentages of non-susceptible *S. pneumoniae *isolates to eight conventional antibiotics to *S. pneumoniae *isolated from CSF and/or blood in eight studies conducted from 1991-2004 are summarized.

The percentage of penicillin susceptible *S. pneumoniae *dropped between 1993-2004; from 71% *S. pneumoniae *penicillin susceptibility in 1993 [8], 63% of isolates were susceptible to penicillin in 2000 [9], to 51% penicillin susceptibility in 2004 (2). The study conducted in 1998-2000 recommended ceftriaxone as the drug of choice for treatment of children with bacterial meningitis [4].

The ES^1999-2000^study identified resistance to Ceftriaxone (84% susceptible) and Ciprofloxacin (82% susceptible) [9]. ES^1998-2004 ^reported 4% multidrug resistance in *S. pneumoniae *[2]. Additionally, ES ^2003-2005^reported that 30% penicillin resistance and 25% erythromycin resistance among the *S. pneumoniae *Egypt isolates [12,22].

The antibiotic abbreviations: PEN, penicillin; OXA, oxacillin; CHL, chloramphenicol; CRO, ceftriaxone; TET, tetracycline; SXT, trimethoprim/sulfamethoxazole; ERY, erythromycin and NA refers to data not available.

(Intermediate resistance is abbreviated as I, and Resistance as R)

One of the initial studies ES^1991-1993 ^that determined the resistance pattern of *S. pneumoniae *was conducted in Abbassia and Embaba fever hospitals. The study involved 1635 children; 961 patients isolates of *S. pneumoniae *were recovered from nasopharynx and blood. Table 1 illustrates the resistance pattern of the blood isolates tested in this study, it is important to note that the nasopharyngeal isolates had similar susceptibility patterns to those of the blood. The study revealed a low percentage of antimicrobial resistance [8]. An increase in penicillin resistance was recorded in a later study conducted by MOHP where 0.8% resistance was detected, however all isolates were susceptible to vancomycin [1]. In 1998-2000 the antimicrobial resistance pattern reported (refer to table 1) recommended ceftriaxone as the drug of choice for treatment of children with bacterial meningitis [4].

A retrospective multicenter study ES^1999-2000 ^conducted in 5 hospitals in Egypt revealed an increase in penicillin resistance, and little resistance to Ceftriaxone (84% susceptible) and Ciprofloxacin (82% susceptible) [9]. A recent sentinel meningitis surveillance program ES^1998-2003 ^showed a marked increase in penicillin resistance (50%-100/205) among CSF isolates in Egypt [7]. Afifi *et al*., reported high rates of multidrug resistance in *S. pneumoniae; *4% of the 206 isolates tested, urging the need to control the dispensing of antibiotic which are generally available as an over-the-counter medication [2]. Additionally, a surveillance report of the ARMed (Antibiotic Resistance Surveillance & Control in the Mediterranean Region) project which started in 2003 and continued for 2 years in the southeastern Mediterranean, reported 30% penicillin resistance and 25% erythromycin resistance among the *S. pneumoniae *Egypt isolates [12,22].

#### Changes in prevalence serotype

In order to assess the efficacy of vaccine and to provide the foundations for the development of effective vaccines for the region it is critical to determine the serotype distribution in Egypt. However, a caveat in such assessment is that isolates tested in the epidemiological studies were from pneumococcal meningitis patients and therefore the identified serotype might not necessary represent all the serotypes causing different forms of pneumococcal diseases as certain serotypes may have a propensity to invade one clinical site. Additionally, variation in the prevalent serotype amongst different studies conducted at different time intervals were shown [7,10,11]. ES^1998-2003 ^study conducted to identify the serotype distribution addressed only pneumococcal isolates obtained from CSF and therefore may not necessarily reflect isolates causing different forms of pneumococcal diseases other than meningitis [7]. A study conducted in Egypt in the late 1970s used Quellung reaction, and identified type 1 as the most frequently observed capsular type [10]. The study by Wasfy *et al*., during ES^1998-2003 ^reported the major serotypes {6B, 1, 19A, 23F, 6A} from CSF specimens of patients with meningitis [7]. ES^1998-2004 ^study by Afifi *et al*., confirmed these finding, the study was conducted during the same time period, and found the same predominant serotypes in Egyptian patients [2]. Additionally 50% of multidrug resistant isolates were characteristic of serotypes 23F, 6B and 6A [2]. These serotypes are inadequately represented in the current 7- (4, 6B, 9V, 14, 18C, 19F, 23F) and 11- valent vaccine (contain four additional serotypes 1, 5, 3 and 7F) and therefore non of the current vaccines are used in Egypt [2,7]. The predominant serotypes reveal enhanced penicillin resistance when compared with other serotypes. Interestingly, Guirguis *et al*., reported in 1990 that serotypes 1, 6A, 9L, 12A, 19A and 29 are the most frequent serotypes identified in the study [11]. These studies impose the importance and urgency to periodically collect and analyze more data on the current predominant serotypes present in Egypt [2]. This will aid in effective treatment strategies, effective vaccine administration and effective means to synthesize new effective vaccines for the region.

### Neisseria meningitidis

*Neisseria meningitidis *was for long reported as the leading cause for bacterial meningitis in Egypt based on studies conducted between 1965-1989 [3,5,13,23], recently it was described as the second or third leading cause after *S. pneumoniae *[1,2,4]. Egypt has experienced several outbreaks caused by meningococcal meningitis serogroup A. During the period from 1966-1989 the country experienced 3 outbreaks with a periodicity ranging from 6-8 years [23].

#### Gradual decrease in the prevalence of Meningococcal meningitis

**Figure 2:**
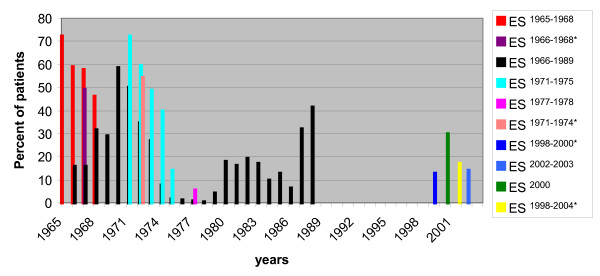
**Percentage of meningiococcal meningitis cases based on studies conducted over 39 years**. Nine different studies were conducted on patients diagnosed with meningitis due to *N. meningitids *from 1965-2004. Between 1966-1989, meningococcal meningitis cases caused by serogroup A are only presented. In ES^1965-1968^conducted on 644 cases of ABM, it was reported that meningococcus was responsible for 56% of bacterial meningitis cases [5]. In ES^1966-1968^on 187 patients, *N. meningitidis *was responsible for 49% of 123 culture positive cases and again identified as the leading cause of bacterial meningitis [6]. The prospective study ES^1966-1989 ^on 7,809 patients also reported that 54% of meningitis cases were caused by *N. meningitidis *[23]. A retrospective study ES^1971-1975 ^done on 1,333 patients reported that 56% of the cases were due to *N. meningitidis *infection [3]. ES^1971-1974^, on 783 patients reported that 54.4% (426) of the meningitis cases were due to meningococcal infection [14]. ES^1977-1978 ^on 1627 CSF specimen demonstrated that *N. meningitidis *was the second most common bacterial meningitis (25.4% of the 350 bacterial meningitis cases) [10]. In the ES^1998-2000 ^on 2047 children less than 6 years, *N. meningitidis *was reported to be responsible for 13% of the 228 bacterial meningitis cases [4]. The ES^2000 ^identified *N. meningitidis *as the second leading cause of bacterial meningitis in Egypt responsible for 30% of the 223 patients positive culture [1]. Prospective study ES^2002-2003 ^on 310 children clinically diagnosed with meningitis detected *N. meningitidis *in 14.2% of the cases and documented it as the second leading cause [21]. ES^1998-2004 ^placed *N. meningitidis *as the third cause of bacterial meningitis [2]. The asterisks represent epidemiological studies reporting an average during the entire period of the study.

Meningitis caused by *Neisseria meningitidis *is referred to as meningococcal meningitis. In early studies from 1965-1968 meningococcus was responsible for more than half (56%) of the cases of bacterial meningitis patients (figure 2). The most affected age group was between 5-15 and the highest mortality cases were reported in ages less than one year. During this 4 years study there was a continuous rise in death rate, which was explained by the development of meningococcal strains resistant to sulpha [5]. Further studies confirmed the prevalence of *N. meningitidis *as the leading cause of bacterial meningitis [3,6,13,23].

*N. meningitidis *possessed a pattern in the occurrence of outbreaks "cyclic nature"; a gradual increase in the number of meningococcal cases every 6-8 years [3,23]. Serogroups A, B, and C were all reported however group A constituted the majority of cases [3,13,14,23]. Most cases occurred during winter months (Jan to Apr) in patients between the ages of 5 and 14. Serogroup A had the least mortality rate compared to serogroups B and C. Mortality was highest in patients less than 1 year and more than 24 years [3]. During the last 2 years of ES^1966-1989 ^an observed change in the clinical presentation of patients with meningococcal meningitis was reported where the onset of the disease was more acute and the course was more severe. Petechial rash was more common in the last years with a higher percentage of mortality occurring in patients developing the skin rash [3,23].

ES^1977-1978 ^studied 1627 suspected meningitis cases and performed CSF analysis; 5.5% of the total suspected cases and 25.4% of the 350 culture-positive meningitis cases were caused by *N. meningitidis*. This was reported as the second most common bacterial meningitis with a mortality rate of 21% [10]. This was the only study during this period that identified serotype C as the predominant serogroup followed by B then A. Most cases occurred from November to April affecting mainly infants and children. Meningococcal meningitis cases had the lowest overall mortality when compared with other bacterial meningitis infections, yet its mortality rate was higher compared to previous studies [10] possibly due to the prevalence of serotype C [3,10].

Later studies showed a decline in *N. meningitidis *[1,2,4,21] and a different dominating serotype. In a study on 2047 children less than 6 years in Egypt ES^1998-2000^*, N. meningitidis *was reported to be responsible for 13% of the 228 bacterial meningitis cases with 23% case fatality. This study reported serogroup B being more common than serogroup A, however, it is worth noting that this was based on 8 isolates tested [4]. A Laboratory-based surveillance of patients with bacterial meningitis was conducted in 14 hospitals from 1998-2004 to determine the etiology and the antimicrobial susceptibility of meningitis pathogens. *N. meningitidis *was responsible for 17% of the 843 cultured positive cases placing it third place after *S. pneumoniae *and *H. influenzae*. When PCR was performed on culture negative isolates the N. meningitidis became the second leading cause constituting 23% of the cases [2]. These studies show a gradual decrease in the prevalence of Meningococcal meningitis and Figure 2 illustrates the percentage of meningiococcal meningitis cases from 1965 to 2004.

#### Antimicrobial resistance and treatment

Resistance of meningococcus to sulphonamides in Egypt was detected in early studies in the 1960s [5,6] and table 2]. The data addressing the resistance profile for *N. meningitidis *in Egypt is sparse owing to the absence of standard criteria for the interpretation of the antibiotic sensitivity data [2].

**Table 2:** Antibiotic resistant *N. meningitidis *isolates.

***N. meningitidis***	**PEN**	**AMP**	**CHL**	**CRO**	**SXT**	**Number of isolates tested**
ES ^1998-2000^ [4]	R = 0%	NA	NA	NA	NA	-8
						-CSF
ES ^1997-2000^ [1]	NA	I = 84%	I = 0%	I = 17%	NA	-48
		R = 2%	R = 0%	R = 2%		-CSF and blood
ES ^1998-2004^ [2]	R = 1%	R = 5%	NA	NA	R = 86	-68
					%	-CSF

The percentage of non-susceptible *N. meningitidis *isolates to five conventional antibiotics to *N. meningitidis *isolated from CSF and/or blood in three studies conducted from1998-2004.

The antibiotic abbreviations: PEN, penicillin; AMP, ampicillin; CHL, chloramphenicol; CRO, ceftriaxone; TET, tetracycline; SXT, trimethoprim/sulfamethoxazole; and NA refers to data not available.

(Intermediate resistance is abbreviated as I, and Resistance as R)

Youssef *et al*., reported that all *N. meningitidis *isolates were susceptible to penicillin [4]. Afif *et. al*., reported high resistance to trimethoprim/sulfamethoxazole, while intermediate resistance was also reported with both penicillin and trimethoprim/sulfamethoxazole in 34% of the isolates. It was also reported that more than 40% of the isolates showed intermediate resistance to either penicillin or ampicillin [2]. Surveillance of antimicrobial susceptibility testing carried by MOHP 1997-2000 on invasive pathogens in Egypt reported that only 2% of isolates were resistant to ceftriaxone [1]. Table 2 summarizes *N. meningitidis *resistance to penicillin, ampicillin, chloramphenicol, ceftriaxone, tetracycline, and trimethoprim/sulfamethoxazole from 1998-2004, note that *N. meningitidis *were isolated from CSF and/or blood.

#### Shift in the serogroups distribution following the implementation of the bivalent A/C vaccine

Early studies indicated that the major serogroup in Egypt was serogroup A, which accounts for 95% of the cases [3,13,14,17]. The observed cyclic nature of the disease reported in the ES^1966-1989 ^study permitted the design of the first successful serogroup A meningococcal polysaccharide vaccine trial in Egypt [3,18]. The results of the first trial conducted in ES^1971-1972 ^indicated that the vaccine conferred significant protection where it was able to produce high degree of immunity for the following three years when compared to the control cases [18]. In 1977 the second clinical assessment for serogroup A meningococcal vaccine was carried out in Alexandria on school children to assess the effectiveness of the vaccine, it was found to be effective for one year following immunization. There was an observed decrease in protection after the first year, which was inconsistent with what was observed during the first trial [16]. A study conducted during the same period 1971-1972 by Sippel *et al*., revealed a high number of carriers of group B and C, anticipating a possible change in the epidemiological pattern of the serogroups in Egypt [15]. A study by Guirguis *et al*., ES^1977-1978 ^reported an unusual pattern in serogroups where C was the predominant followed by B then A [10].

In 1992 the MOHP initiated a school based vaccination program with the bivalent A/C capsular polysaccharide vaccine. The vaccine was taken at age of 6 years followed by a second dose 3 years later. A retrospective review by Nakhla *et al*., to understand the change in epidemiology of meningococcal serogroups after the introduction of the bivalent vaccine indicated a significant decrease in meningococcal cases following the implementation of the school-based vaccination program [17]. It is worth noting that the expected outbreak in 1996 has actually never occurred and it was reported that there is a decline in the incidence of meningococcal meningitis cases [23,24].

A more recent study ES^1998-2000 ^reported a higher number of serogroup B than A [4]. Another study by Afifi *et al*., reported out of 135 isolates of *N. meningitidis *51% were group B, 35% group A, 4% group W-135 and 2% belonged to group D and Y [2]. The project conducted by MOHP in 2000 in addressing the communicable diseases in Egypt reported that meningococci were compromised of 54.5% of serogroup B, 31.8% group A, 4.5% group W135, 2.3% group Y [1]. This shift in the serogroups was observed after the implementation of the bivalent A/C vaccine by MOHP implying the significant impact that the vaccine had on the epidemiology of the disease [17].

### Haemophilus influenzae

Sparse epidemiology studies are available addressing the pathogenicity of *H. influenzae*. It was less implicated in meningitis in earlier studies conducted in Egypt [3,13], yet recently it was reported in limited studies to be one of the leading causes of bacterial meningitis in Egyptian children [1,2,4]. It is difficult to estimate the incidence of *H. influenzae *meningitis in Egypt due to absence of population based analysis and limited surveillance studies [4]. One study ES^1998-2004^, defined *H. influenzae *serotype b as the main cause of pneumonia in children below 5 year [2].

#### *H. influenzae *the leading cause of meningitis in very young children

A prospective study ES^1966-1989 ^on 7,809 patients admitted to the Abbassia Fever hospital, reported *H. influenzae *to be the cause of meningitis in 322 of the patients (4.1%) who had a mean age of 2.5 years; it is worth noting that most of the cases (76%) were patients less than one year. Most cases occurred in winter months with a gradual increase in the number of cases from one year to the next. The mortality rate of *H. influenzae *was 39% [3,23]. In the two-year study ES^1966-1968 ^conducted at AFH on 187 patients, *H. influenzae *was responsible for 12% of 123 culture-positive cases identified [6].

A retrospective review of 1,333 patients with acute bacterial meningitis admitted to the Abbassia Fever hospital from 1 January 1971 to 31 December 1975 reported only 2.6% (35 cases) of the cases were due to infection with *H. influenzae*, and most cases were children less than one year (57%) with 27% case fatality [13].

Following this another two year study 1977-1978 at Embaba and Abbasisa Fever Hospitals *H. influenzae *was reported to be responsible for only 2.6% of the total 1627 CSF specimens and 12% of the 350 culture positive isolates. In contrast to the low frequency of *H. influenzae *infection the mortality rate was high (57%) among very young children with type b [10].

**Figure 3:**
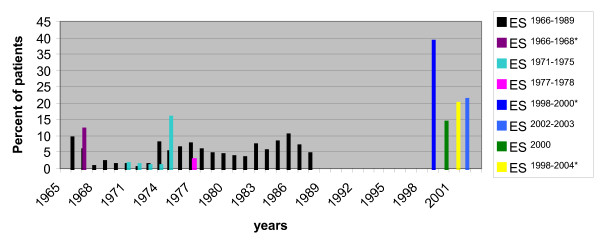
**Percentage of Meningitis Cases Caused by *Haemophilus influenzae***. Eight different studies were conducted on patients diagnosed with meningitis caused by *H. influenzae *from 1965-2004. ES^1966-1989 ^reported *H. influenzae *to be the cause of meningitis in 4.1% of 7,809 patients [3]. In ES^1966-1968 ^*H. influenzae *was responsible for 12% of 123 culture-positive cases [6]. ES^1971-1975 ^study of 1,333 patients with ABM reported only 2.6% of meningitis cases were due to infection with *H. influenzae *[13]. ES^1977-1978 ^*H. influenzae *was reported to be responsible for 2.6% of the total 1627 CSF specimens and 12% of the 350 culture-positive isolates [10]. ES^1998-2000 ^on children less than 6 years, 39% of the 228 patients' cultured- confirmed disease 39% showed *H. influenzae *[4]. ES^2000 ^revealed that *H. influenzae *was responsible for 14.3% of 223 positive bacterial culture meningitis cases [1]. ES^2002-2003 ^on 310 children clinically diagnosed with meningitis identified 202 cases as ABM where 21% of the ABM cases were caused by *H. influenzae *[21]. ES^1998-2004 ^*H. influenzae *was responsible for 20% of the 843 cases of culture-positive patients [2]. The asterisks represent epidemiological studies reporting an average during the entire period of the study.

Surveillance for patients with bacterial meningitis in 12 hospitals in Egypt between May 1998 and December 2000 was conducted to determine the etiology of the disease in children less than 6 years. Of 228 patients that had cultured- confirmed disease 39% showed *H. influenzae *as the leading cause of the disease with 81% of the *H. influenzae *case-patients were less than 12 months. The mortality rate was 27% with serotype b being responsible for most of the cases [4]. As a part of a project performed by the MOHP in Egypt, it was reported that *H. influenzae *was responsible for 14.3% of 223 patients having bacterial meningitis [1]. A prospective study ES^2002-2003 ^conducted in Alexandria fever hospital on 310 children clinically diagnosed with meningitis, detected 21% of the cases were caused by *H. influenzae*, again the leading cause of acute bacterial meningitis in this study [21]. In a laboratory-based surveillance ES^1998-2004 ^carried out to identify the etiological agent of bacterial meningitis, *H. influenzae *was found to be the second leading cause following *S. pneumoniae *responsible for 20% of the 843 cases of culture positive patients. Among children less than 5 year *H. influenzae *was the most common bacteria isolated. The mean age was 7 months with a case fatality ratio of 25% [2]. Figure 3 illustrates the prevalence and trends of *H. influenzae *in bacterial meningitis cases.

#### Antibiotic resistance and treatment

The study by Ostroff *et. al*., is considered the first study in Egypt to identify the resistant pattern of *H. influenzae*. Although no resistance was detected in the isolates obtained from the blood, yet strains obtained from 347 nasopharynx isolates conferred certain level of resistance for the tested antibiotics. It was reported that 1.4% and 5.6% of the isolates conferred intermediate and full resistance to ampicillin respectively, 10.8% and 4.3% conferred intermediate and full resistance to SXT respectively, 1% and 10% conferred intermediate and full resistance to chloramphenicol respectively (table 3). The yield of isolates from blood as indicated in table 3 was very low (only 6) to be able to draw definitive conclusions regarding the resistance pattern [8].

**Table 3:** Antibiotic resistant *H. influenzae *isolates.

***H. influenzae***	**PEN**	**AMP**	**CHL**	**CRO**	**TET**	**SXT**	**-Number of isolates tested** **-Source**
ES ^1991-1993^ [8]	NA	I = 0%	I = 0%	NA	NA	I = 0%	-6
		R = 0%	R = 0%			R = 0%	-Blood
ES ^1997-2000^ [1]	NA	I = 16%	I = 24%	I = 0%	NA	NA	-75
		R = 30%	R = 60%	R = 6%			-CSF
ES ^1998-2000^ [4]	I = 0%	I = 16%	I = 20%	I = 0%	I = 5%	I = 5%	-47
	R = 45%	R = 63%	R = 67%	R = 0%	R = 81%	R = 45%	-CSF
ES ^1998-2004^ [2]	NA	R = 45%	R = 37%	NA	NA	R = 40%	-119
							-CSF

The percentage of non-susceptible *H. influenzae *isolates to seven conventional antibiotics to *H. influenzae *isolated from CSF and/or blood in three studies conducted from1991-2004.

The antibiotic abbreviations: PEN, penicillin; AMP, ampicillin; CHL, chloramphenicol; CRO, ceftriaxone; TET, tetracycline; SXT, trimethoprim/sulfamethoxazole; ERY, erythromycin and NA refers to data not available.

(Intermediate resistance is abbreviated as I, and Resistance as R)

The Surveillance conducted by MOHP reported increase in the resistance pattern from the above study. It also reported multi-drug resistance (TET, SXT, and CHL) in 29.6% of the isolates, in addition to positive β-lactamase activity [1].

Youssef *et. al*., demonstrated that less than 50% of *H. influenzae *isolates were susceptible to chloramphenicol and ampicillin while all isolates were susceptible to third generation cephalosporins; ceftriaxone. It was reported that higher mortality rate were found in patients with *H. influenzae *resistant isolates to AMP and CHL [4]. In the laboratory-based surveillance ES^1998-2004 ^conducted in a network of 14 hospitals 45% ampicillin resistant isolates were reported, 37% of which demonstrated beta-lactamase production (Table 3) [2].

#### Serotype distribution and vaccination

Since earlier studies *H. influenzae *serotype b was predominantly reported among the few reported cases. Guirguis *et al*., reported that all *H. influenzae *isolates were serotype b; out of 47 *H. influenzae *isolates 30 were tested and all of them were type b [4]. The finding that the majority of *H. influenzae *isolates were type b [20] and a common cause of acute lower respiratory infection allowed the introduction of Hib vaccine by the MOHP in the EPI (Expanded Program on Immunization) [1,4].

## Concluding Remarks

The dominating causative agent for bacterial meningitis has changed over the forty years period studied. Studies have demonstrated that Pneumococcal meningitis is currently the leading cause of meningitis in Egypt. Studies on the prevalent bacterium in different age group shows that *H. influenzae *is the leading cause of meningitis in very young children. The serotype prevalence of some bacterial species were altered over the years changing the mortality rate of the disease. Several bacteria have developed antimicrobial resistance to conventional antibiotic regimes and emerging multidrug resistance strains. The referenced studies have provided the platform for assessing the prevalence and antimicrobial resistance pattern of bacterial meningitis in Egypt. However, this also iterates the need for periodic and continual surveillance to guide the empirical treatment of such diseases.

## List of Abbreviations

ES: Epidemiological Study; AFH: Abbassia Fever hospital; MOHP: Ministry of Health and Population; EPI: Expanded Program on Immunization; PEN: penicillin; AMP: ampicillin; OXA: oxacillin; CHL: chloramphenicol; CRO: ceftriaxone; TET: tetracycline; SXT: trimethoprim/sulfamethoxazole; ERY: erythromycin; ABM: Acute Bacterial Meningitis.

## Competing interests

The authors declare that they have no competing interests.

## Authors' contributions

LS reviewed the literature, analyzed the data and wrote the preliminary draft and RS conceived, designed and coordinated the study and edited the draft into the final version.
